# Quality of Life Philosophy V. Seizing the Meaning of Life and Becoming Well Again

**DOI:** 10.1100/tsw.2003.105

**Published:** 2003-12-01

**Authors:** Søren Ventegodt, Niels Jørgen Andersen, Joav Merrick

**Affiliations:** The quality of Life Research Center, Copenhagen, Denmark

**Keywords:** quality of life, QOL, philosophy, human development, holistic medicine, public health, Denmark

## Abstract

This paper presents a positive philosophy of life developed to support and inspire patients to take more responsibility for their own lives and to draw more efficiently on their known or hidden resources. The idea is that everybody can become wiser, use themselves better, and thus improve quality of life, subjective health, and the ability to function.To be responsible means to see yourself as the cause of your own existence and state of being. To be the one who forms your own life to your liking, so that others do not shape it in the way they prefer to see you. Seen this way, taking responsibility in practice is one of the most difficult things to do. One of the greatest and most difficult things to do in this context is to be able to love. To be the one who loves, instead of being the one who demands love, care, awareness, respect, and acceptance from somebody else.Since almost all of us have had parents who maybe loved us too little and mostly conditionally, we all harbor a deep yearning to be loved as we are, unconditionally. A lot of our energy is spent trying to find recognition and acceptance, more or less as we did as children from our parents, who created the framework and defined the rules of the game. But today, reality is different. We have grown up and now life is about shaping our own existence. So we must be the ones who love. This is what responsibility is all about. Taking responsibility is, quite literally, moving the barriers in our lives inside ourselves. Taking responsibility for life means that you are willing to see that the real barriers are not all these external ones, but something that can be found within yourself. Of course there is an outside world that cannot be easily shaped according to your dreams. But a responsible point of view is that although it is difficult, the problem is not impossible; it is your real challenge and task. If there is something you really want, you can achieve it, but whether it happens depends on your wholehearted, goal-oriented, and continuous attempts. This paper describes the philosophy about seizing the meaning of life and becoming well again, even when there is little time left.

## INTRODUCTION

To be healed means to become whole again. The only thing that really can make us whole is to let go of everything in us that exists as separate and unconnected parts. The fragmented way we describe reality in our minds fragments our existence and makes us lose our innate coherence[1]. The egotistic nature of our philosophy of life makes us lose our innocence, our spiritual purity, and the loving, sound, and honest quality of all our inward and outward relations[2].

The marvelous thing is that we can get our life back[3]. No matter how much dirt we have collected, no matter how much trouble we are in, now matter how much negative we have done, we can get our life back. But that is only if we are willing to clear up the mess. The cure to become well again, to get back to life, and become whole again is the same whether we are ill physically or psychologically, lonely, or suffering from existential problems that make us contemplate suicide or make us unfit for work. This is what the medical arts and all real healing are about.

If you become ill — and now we talk about diseases like cancer or a stroke, arthritis, alcoholism, depression, or even light degrees of dementia — it seems that you can reclaim your life if you are willing to fight really hard to keep it. But you must start the fight here and now. If you want healing, you want it today and you want to fight, but it can be that you do not know how to do it.

We believe that usually the individual is able to do much more for him- or herself than any physician, nurse, alternative therapist, social worker, or any other expert can do. Only people who fight the fight of their lives and invest their total resources in themselves stand a chance of getting their lives straightened out again.

## HOW DO YOU FIGHT FOR YOUR LIFE?

Here is the $100,000 question: How do you fight for your life? How do you revitalize your whole existence? How do you regain youth, life energy, and joyfulness? The reason that so few people these days have any success with this project is that it is paradoxical: The fight is the fight for not fighting. The fight is a fight to let go. The fight is a fight to bring all the futile efforts and worries to an end and be relaxed, present, natural, easygoing, focused on the good things, and balanced.

What make us ill are all the life-denying decisions we take when we are overwhelmed by pains and troubles[4]. The reason that we take these decisions is that by doing so, we can escape pain. The pains of the situation are suppressed when we choose to describe reality in a way that minimizes our own responsibility. This is very simple, but generally poorly understood. So, what is required for healing is letting go of all the limiting beliefs, all the life-denying decisions we have taken though our lifetime. The minute we do that, we start to heal. The reason that we have to fight doing this is that our philosophy of life lies embedded in pain and only through confronting and reliving that pain can we get so close to the decisions that we can actually articulate them precisely enough to let go of them.

The sick or troubled person who leaves the responsibility for the essential decisions in life to others can be certain that the decisions made are not optimal. This will lead the person to go downhill for as long as this lack of responsibility continues.

To demand to be in authority of your own life, to be the one to make decisions within your own personal circle, is to become totally independent. You have to grab hold of your own brave lion's heart and say "yes" and "no" in life. Do you live with your heart or have you already pawned your soul? To regain a personal and independent firsthand knowledge of life and the world is what is needed in your fight back to life.

Only a person who really believes in him- or herself has the opportunity to discover what is really right or wrong for him- or herself. If you do not have the courage to listen to your inner self, you will inadvertently live a life in which you will have to rely on others' interpretation of things, especially when life is difficult. Think of life as a great and complicated building. Every right decision throughout life adds a stone to our life's castle. Every small wrong choice removes a stone from the castle. If the balance tips the wrong way we will gradually, over the years, lose the order in our lives, tear our building down, and gradually but systematically become more disconnected and superficial.

If we are observant, we will see how our organism gradually falls apart and degenerates towards death during the last decades of our lives, our old age. Old age and sickness is the result of hundreds of life-denying decisions that you now have to identify and let go of to be healed. This is not, as you might expect, an intellectual effort. This work is done through our feelings. That is the big trick: to feel, to understand, and to let go.

Concurrent with ruining or trivializing our social relations we become poorer in our self-expression, less sufficient, and have fewer resources. Concurrent with our resigning ourselves as far as our dreams are concerned, our self-confidence suffers a serious break. Concurrent with our losing our relationships with the surrounding world, we lose the feeling of value and self-worth while our inner selves gradually lose the experience of coherence and the meaning of life.

The poor condition so many old people experience is neither natural nor unavoidable. If you ask yourself whether you are on the way up or down you will see what we mean. It is vitally important to your future life that you are on your way up throughout life. From the start many people abandon such ideas, but it seems to us that somewhere they have given up already. To us it is essential to believe that life keeps getting better, more faultless, and richer on energy and more balanced. To us it is this outlook that characterizes those who do their best to live wholly throughout life.

If you recognize the downward pattern in your life, the great trick is to change this pattern so that life systematically becomes better again, so that you become well again if you happened to be ill. The day you stop collecting negative beliefs and start to let go of your stock of negative beliefs, the magic happens. You see, it is not that difficult really. But you must find the key. We are meant to be cheerful and lighthearted, and if we are not, today we can begin to change to become so.

In theory, the recipe is very simple. Acknowledge your misconceptions of life, yourself, and the world and adjust your map by letting them go. Open yourself humbly to the fact that you have not understood the most profound and important things in life and seek high and low for the meaning of life. With the right spirit this project always succeeds as long as there is enough time.

Oh, we better add that there is never enough time. But usually, the awareness of a definite destruction and death that invariably comes closer is very motivating. Since we all have to die in a little while and still carry on living as if we have oceans of time, awareness of death is something we are rarely open to.

## BEING ILL AND WANTING TO BECOME WELL AGAIN

Most people react to serious diseases by becoming scared, maybe apprehensive, and often terribly unsure. What is happening to me? Am I dying? Will I ever get well again? Will my life be the same? The majority of people visit the physician when they feel really sick. It is important to know what you are up against, what the physician can say about what is wrong with you. It is important not to be to shy when you talk with the physician, otherwise you will not get an answer to all your questions. Remember to ask for referrals to the specialist whom you feel you need to consult in order to get a proper diagnosis.

It is a great art to use the physician as a consultant that provides you with the information you need to take the right decisions. The reason you cannot just receive a diagnosis and a treatment is that it is your life, so you must stay responsible, knowing what is going on and using your own beliefs and understanding of the healing process as your guidelines.

Of course, there is usually a lot of sense in what the physician is saying. Modern physicians are experts in diagnostics. But healing, on the other hand, is not just about sense. It is more about how we come to experience our life, body, and soul. Major changes in perception and experience often lead to major changes in the rate of healing.

It is our claim that the cause of most diseases today is unknown to medical science. Therefore, the physician probably does not know the reason for the illness you are consulting him about. A physician can treat you in accordance with tradition and good practice, but he is not the one who heals you. Today a physician usually mostly treats symptoms. He can describe what usually happens with a given treatment and in this way he can make a prognosis. But the prognosis is only valid if you are an ordinary patient, a being who accepts that the physician is responsible for the treatment. You cannot do that if you work with yourself. In his book *Love, Medicine and Miracles*, the American surgeon Bernie S. Siegel called this latter type of patient “exceptional” and he discovered that they fare much better than the average patient[5].

When you start taking responsibility for becoming well again by systematically and industriously finding and letting go of life-limiting beliefs, you create a new balance within your life. From this day on you belong to a group that is not covered by ordinary statistics.

It is our hope that over the next 10–20 years, surveys like The Danish Quality of Life Survey will provide us with useful new statistics and prognoses for those patients who struggle with their views of themselves and the world, and in this way obtain a better quality of life.

When you are ill, you are often scared and unsure and in that state it is terribly difficult to say no to something your physician proposes, even if you do not really believe in it. He can talk about an operation or chemotherapy, some medicine with heavy side effects, or perhaps a hormone supplement that you would rather do without. We can only recommend that you do what you know, deep down, is right after you have collected all the relevant information. Sometimes the physicians will think you are crazy and maybe they are right, but it is your life and you are the one who is responsible and therefore must take all the decisions yourself. Do not let go of the controls! Let go of the mistrust and the negative decisions.

## TAKE RESPONSIBILITY

To take responsibility and shape your own course through illness is often your only chance for survival, because modern physicians do not cure most of the serious diseases. You often carry them with you through life. So do not let your life be a continuous battle against symptoms that medicine cannot really suppress. Be brave-hearted and save yourself by assuming responsibility for your life. Feel the pain, understand your past, and improve your philosophy of life on a daily basis, every single day letting go of still more negative attitudes to life. This will make your quality of life and health go straight up like a hot-air balloon.

Life is rough and much courage and wisdom are needed when you are suffering from a serious illness, like keeping your balance on a thin tree trunk spanning a chasm. There is no way back, so you better not fall down. To relinquish responsibility makes you inattentive and controlled by somebody who does not have your opportunity to see what is happening. Be awake and be present if you love life.

It is easy to have a strange relationship to that part of the body that is hardest hit by the disease. “It is not me at all, I am not like that!” “It is not my body, my stomach, my leg, or my breast that looks like that or feels like that.”

Distancing yourself from the problem is an understandable reaction. The distance and resignation immediately remove some of the suffering. But looking at your body and illness in this way, you help to lock the situation in place and perhaps push developments in the wrong direction. To be able to really want to become well again you have to confront the suffering of being ill. You need to carry this suffering into your very soul. If you distance yourself from the suffering, you often also distance yourself from getting cured.

We do not mean to say that you have to devote yourself to the suffering, but you need to turn towards it and not away from it, so to speak. Feeling the pain associated with the sick part of the body is often the key to healing because that pain represents the relevant, life-limiting decisions you took in the past. Confronting the pain makes these decisions surface in consciousness where they can be analyzed, articulated correctly, and finally released — dropped for good, leaving you with a better attitude towards life.

To be healthy and well means that the body, the whole organism, is full of life. A healthy body is a direct manifestation of our innate wisdom and vibrates with all our vital energy. Basically, the body is a manifestation of life's innermost recipe for a human being. We are meant to love our own flesh, to be totally present in it and accept it and live it. To be present is an absolute necessity for being totally healthy and well. When you are ill, the disease often takes up a great amount of space in your life. The disease can monopolize all available resources and all our mental energy. When we are ill, we easily become sad and depressed. We feel sorry for ourselves and we easily end up in a vicious circle where life can go dramatically downhill before we know it.

However difficult, the only way to get well is to be occupied by something else. This does not mean that you must lie to yourself about your illness or run away from it, not at all. It means that at some deep level within your life the disease may be connected to some basic suffering or imbalance that needs to be faced. The existential problems that are the real cause of the disease appear as your existential pains and your worried mind. In order to become well you need to turn your attention away from all concrete aspects of disease and turn towards its roots, the underlying reasons — the pains and the life-denying decisions you took — to make them go away.

In our opinion, this process of healing and learning will always lead you to the center, to the core of existence, and to the suffering you feel here, when life is not what it should be. A deep existential distress is inevitably found and felt. A terrible old pain, which made you abandon the very meaning of your life, often many years ago. To us the project of becoming well again is identical with the project of discovering the profound meaning in and joy of life. As soon as you let go of the negative decisions, the joy and vital energy will come back.

## JOY OF LIFE

As explained in the previous articles of this series, it is our opinion that we become ill if we do not retain this joy in our consciousness and carry it with us throughout life. Forced by necessity, therefore, our chance of becoming well again is dependent on our rediscovering this profound joy of life. It is hidden behind negative decisions that are hidden behind existential pain that is hidden behind our basic denial of our life as painful and superficial.

How is this done? Basically and in principle it is very easy: feel, understand, let go of your limiting beliefs. Unfortunately, one cannot provide a specific recipe that every ill person can follow. It is easy to give advice, but when it comes down to it, it is rarely worth much as everybody must find out what to do for him- or herself.

Health magazines are overflowing with advice mostly about health-improving behavior. They seem often fair and relevant, but can be so difficult to follow that you just have to smile. Here is a list of examples:

> *"Every morning and night for 10 minutes think of two nice events in your life. Make sure that you laugh for a total of 30 minutes before lunch. Try to have a confidential conversation every day with at least one person who means something to you. Tidy up your personal relationships and your past. Tell yourself 400 times a day that you love life, accept it as a challenge, and dig out your love for life. Eat exactly what you want when you have discovered what you need. Drop sex and be together in love, there are more important things to use your energy on. Find out why you became ill. Keep asking yourself until you have an answer that you can use to help yourself to become well again. Use death as a guide; each night in the dark before you go to sleep try to feel like a corpse and see that you change the cold, black feeling to a feeling of life and warmth so that you feel totally alive before you fall asleep. Start dreaming that you will get well again*

> *and then dream about what is needed to achieve this goal. Trust the profound wisdom of life and do what you feel you need to do even though it does not make sense to you."*

To start with, the person who possesses the self-discipline and energy to follow all this advice is not likely to become ill in the first place. Second, the key to improving your quality of life and thereby your health is more likely to be emotional than behavioral. Your basic problems in life are about emotional pains and understanding, not primarily about behavior. Good behavior will eventually follow fine understanding, but just doing without being will not take you there.

Although in the middle of all the confusion, concrete advice like this may appear to be the right place to start, it is often quite misleading and will quickly do more harm than good. You can easily waste what little energy you have. It is up to the individual to discover what needs to be done in order to get back to life, and to feel deep inside that what is being done is the correct thing. You must have the feeling that it is kill or cure and that you can do nothing else.

Allow us to make a couple of general suggestions. Please remember that they reflect our personal view of life and disease and may be wrong for you. The difficulty in becoming well again seems basically to be about creating your own life. Some day you are well again and acknowledge that you have recovered. In reality, this acknowledgment covers a profound, existential decision that you are well again. But the problem is that you cannot make such a decision just like that, it needs self-confidence and lots of personal energy, which you actually do not have just now. If you believed that you had this possibility you would no doubt just use it! It is not really about deciding to be well, but to let go of all the negative decisions that made you ill.

Let us start with the perspective that what you lack is some energy. As we suggest that you do not focus on the disease, but on the basic existential problems, we shall talk no more about disease, but turn towards the all important: reclaiming or rediscovering the meaning of life.

## BECOMING MORE ENERGETIC

One fruitful method of thinking about personal development is by using the concept of personal energy (please note that we are dealing here with "energy" as experienced, not physical or chemical energy). With its energy, the living organism can carry out a piece of work, like influencing the world or changing the state of the organism, quite analogous with the energy in a battery, which can drive an engine or an electric bulb. The biological system possesses something we have every right to call the energy level: our personal energy, high or low.

Now, what is the personal energy? From where does it come? And how can we lose it? It seems to us that vital energy stems from knowing your own personal meaning in life, and that we lose it by denying it as a means to escape emotional pain. It is the meaning of life that makes people make a real effort and fight persistently and continuously to realize their dreams.

Knowing the meaning of your life is just something that opens the gates. The energy stems from life itself. And you can lose it if you live contrary to the lawfulness that applies to human beings. Sometimes we feel that we bubble with energy and strength. At other times we feel down, weak, and depressed. By thinking of your condition as an expression of a low personal level of energy, it becomes possible to analyze the situation in detail: What decides whether my level of energy is high or low?

How do we waste our energy? Think about the people you have met during the day. How many did you argue with about some insignificant detail? What happened? A brief contest of wills? The confrontation was not free, was it? You wasted your vital energy on the confrontation and to no avail. And what about the argument with your partner at the breakfast table? What about your self-pity, when you did not win the small photography competition? Why did you not say no to the little extra job your boss asked you to do, and which you really did not want to do? All these episodes throughout the day where you were not completely honest but compromised with yourself or even let yourself down? What about the time when you needed a break, but did not take it because you wanted to please a colleague? What about your worries about tomorrow because you probably will not finish the project as promised? And so it goes on. Viewed like this, it is obvious that most of us live a life out of balance, and we cannot seem to use the energy only when it is wise and right.

A very interesting aspect of the personal energy level is humor. Some people are always having fun and they cannot help it. We are not talking about foolish and vulgar jokes overheard in locker rooms, but about real, spontaneous humor brought forth in the situation, because people just cannot help themselves. People who are down are also without humor. Just think of sick people. They rarely make spontaneous jokes. People who have retained their sense of humor and can laugh at themselves and their often somewhat comical situation encased in plaster or bandages or whatever, are often the ones who become well again. *Anatomy of an Illness as Perceived by the Patient* is the title of a popular book by Norman Cousins[6], who describes in great detail how he survived a malign autoimmune disease that attacked his body's connective tissue, but survived despite the death sentence of his physicians. His strategy is to be recommended. There is hardly any doubt that if you can regain your high level of vital energy, as it is expressed in humor, you are often able to become well again.

To live here and now, to be healthy and well, presupposes a high personal level of energy. The correlation between a personal high energy level and illness can be explained in a simple way, if we see illness as an expression of a collapse in the biological order of the organism. We become ill when our personal level of energy is too low, because the experienced level of energy is closely connected to the inner order of man's organism. When we experience that the energy level as a whole is low, intercommunication between the cells is poor. Then the organism's energy level is too low and the cells are no longer able to receive the information needed to do the right thing, where they are supposed to do it, and thus maintain a healthy organism. Therefore, when the energy level rises, your health becomes better and vice versa.

The good life and a high quality of life require energy and we just cannot afford to waste it on foolishness. It is not too much to maintain that the ability to think in a complex way and really penetrate into your existence and understand it is directly connected with the energy level. When we are down, we are functional fools without the ability to understand what is going on around us or to make the choices needed to create the good life for others and ourselves.

Therefore, to keep account of our energy is an absolute necessity for each and every one of us. To take stock throughout the day of our use of energy and to find out whether we are wasting it and where we obtain something of real value.

## ILLNESS, SYMBOLS, AND MYSTICISM

You can also understand and tackle your illness in a symbolic way. What you fight, then, is material that has been poorly integrated into the subconscious, and which manifests itself in various symbolic ways. A symbol is a kind of picture that is loaded with meaning. The symbols can be said to represent the fundamental concepts to be found in human consciousness, where the description of the world meets life itself.

Symbolic expressions of imbalance can be identified through dreams, Tarot cards, I Ching symbols, astrological or alchemical symbols and interpretations, mystical rituals, or any other way you prefer to enter the symbolic universe. Unfortunately, the great problem with a symbolic approach is that everything quickly becomes mysterious and strange. Although life really is a profound mystery, in our opinion a mystifying mood is not very conducive, because a mystery actually means that our minds do not understand life and reality.

If we see this clearly and step back from understanding the world in a rational way and just look at it with our heart as suggested by Antoine Marie Roger Saint-Exupéry (1900–1943) in *The Little Prince*[7] (where the fox teaches the crashed pilot in the desert), then life becomes simple and easy to understand. In a way, the mysticism arises because we insist on bringing our mind along into the irrational dimension. Jesus talked about finding the truth by becoming like a child again. Children do not have much flair for mysticism, which is reserved for the adult. But this is all too often nourished by self-pity: "Poor me, I have never understood life" or an almost opposite feeling, that is, self-importance: "Oh, I am really chosen to save the world thanks to my mystical experiences."

Try to confront a small child with a dead bird or a dead person and rejoice in how simple and direct the child sees the situation and reacts to it. And then compare the child with the adult holding the child's hand. Notice how strangely complicated and almost alien death and the whole of life have become to us. Well, if you have a penchant for mysticism you must differentiate between life-giving and life-taking symbols and place yourself on the side of the life-giving symbols. In practice this is a great achievement that demands a lot of energy, and it may even require you to consciously enter your dreams to untangle the threads as Carlos Castaneda, inspired by the Native Americans, suggests in the book *The Art of Dreaming*[8]. *The Tibetan Book of the Dead*[9] may also be of help (Timothy Leary has written a simple introduction[10]), and while some people benefit from the classical Chinese science book *I Ching*[11], it is not so easy to understand. The *I Ching*, or *Book of Changes*, is the most widely read of the five Chinese Classics. The book was traditionally written by the legendary Chinese Emperor Fu Hsi (2953–2838 BCE).

In our opinion, the world of symbolism requires a very high level of energy and it is fraught with danger of becoming lost. So perhaps the world of symbols is not so attractive after all. Mysticism often invites you to escape from reality instead of taking the action needed to get you out of the morass. The mysterious mood is driven out by a clarified mind, although it must be admitted that nothing in life is quite as clear and simple as you may think life and the world is in a moment of clarity. Far too many people have read about mysteries and they are helplessly stuck and never get to experience life's mysteries in their own lives.

## TO TAKE RESPONSIBILITY AND MOVE BARRIERS

It is easy to agree that we have to take responsibility. But what does it really mean? To be responsible means to see yourself as the cause of your own existence and state of being. To be the one who forms your own life to your liking, so that others do not shape it in the way they prefer to see you.

Seen this way, taking responsibility in practice is one of the most difficult things to do. Just think of how often you argue with your partner or your best friend. What is needed to stop arguing or just make the arguments less frequent and less violent? It is not difficult to acknowledge that if one of you stopped arguing, the other person would not have a chance. One cannot argue alone. It is your responsibility. But why should I start by being magnanimous and forgive my partner his or her flaws? Why should I start being the one to make allowances and keep the status quo? The answer is that in reality the greatest and most difficult thing to do is to be able to love. To be the one who loves instead of being the one who demands love from somebody else.

Since almost all of us have had parents who loved us too little and mostly conditionally, we all harbor a deep yearning for being loved as we are, unconditionally. A lot of our energy is spent trying to find recognition and acceptance, more or less as we did as children from our parents who created the framework and defined the rules of the game. But today, reality is different. We have grown up and now life is about us shaping our existence; we are the ones who love. This is what responsibility is all about.

Taking responsibility is, quite literally, moving the barriers in our lives inside ourselves. When we do not take responsibility for life we experience that all our problems, everything that prevents our dreams coming true, are caused by outside obstacles: our inadequate wages or salaries; the terrible flaws in our friends, partner, or boss; unemployment; expensive colleges; friends who let us down; or accidents like car crashes and sudden illness.

Taking responsibility for life means that you are willing to see that the real barriers are not all these external ones, but something that can be found within yourself. Of course there is an outside world that cannot be easily shape according to your dreams. But a responsible point of view is that although it is difficult, the problem is not impossible; it is your real challenge and task. If there is something you really want you can achieve it, but whether it happens depends on your wholehearted, goal-oriented, and continuous attempts.

## BECOMING SELF-CONFIDENT AND WISE

When you believe in yourself you are able to listen with your inward ear. You can listen for what you really need, deep down. Actually, it is something of a mystery that this process makes us wiser, because how is wisdom stored in our soul, so that those who pay attention to their intuition or inner voice, or whatever you choose to call it, become wiser than the ones who do not? And how do you achieve self-confidence in the first place?

You could say that self-confidence is something we acquire through our victories. But as self-confidence also is what makes us act wisely, which leads to success, we have not learned much from this. On the other hand it is obvious that those who succeed in the long run are the ones who keep fighting for what they love. They do it in such a way that defeats are mined for learning and insights, which improves their chances when the next struggle comes along. It is clear that people with the ability to humbly keep fighting and learning from their defeats and victories are the ones who succeed in the long run.

But from where does this fundamental toughness come? It comes from sensing that life has a profound meaning and living accordingly. If you intensely experience the meaning of life and know what you like deep down, where you want to go, and which dream you want to fulfill, you will keep fighting despite failures. Never giving up and always being open to learning will increase your chances of getting there. You may call this being a fighter, a warrior, or just being strong-minded. Those who keep fighting are the ones who have acknowledged that they themselves are the enemy. Therefore, the only way out is to take up the challenge and fight life's hardest battle, that is, the battle against yourself or rather all the flawed and peculiar things we believe about life and ourselves. Paradoxically, to win over yourself is to let go of your misconceptions, but this letting go on the contents of the ego seems to be the hardest thing one can ever do.

Why are the ones who believe in themselves wiser than the ones who do not? The classical and good explanation is that life on its own holds enormous wisdom, which our limited reason, our mind, does not really know or acknowledge. Therefore, when we listen to our innermost voice we have millions of years' experience to draw from, experience that has been gathered by living matter, stored and carried forward through time, ready to be taken out and used when needed.

How can we know that what we sense and feel is also the real thing? Certain knowledge about life is a result of sophisticated reason attuned to well-developed intuition. Our reason needs to feature a good image of life, the world, and ourselves as a living soul. An image of ourselves as victorious, successful, and lucky in carrying through what we set out to do and purified of our defeats in life. Intuition must be purified of all the feelings contained in previous experiences so that the experience of reality is totally up-to-date and true.

If you turn your consciousness towards life itself in an active process where you listen and make an effort to understand what the peculiar wordless voice whispered by life itself tells you, then you can draw from the enormous knowledge and wisdom about life. All of life's great and important occurrences are already well described in general and superior ways and can be drawn down to earth and used through our dreams. The great dream about love, for instance, or the dream about a great friendship, meaningful employment, or really good relationships with everything here on Earth.

During puberty, when we meet a suitable person of the opposite sex, these intense dreams about sex and love start pouring in. They come from nowhere and suddenly they fill everything. Actually, the source is very insistent because it is this intuitive knowledge that is the basis of our whole existence, all the things in life that really mean something to us.

Still, we cannot escape the struggle involved in finding out what we actually need to do in every situation throughout life. What to say to our first partner can be a real problem and how to behave at our first place of work can be a real challenge. We are at a loss and must solve all the problems on our own. However, our inner nature possesses overall and abstract guidelines that can lead use safely through life.

## TO USE DEATH AS A MIRROR

Death is the real enemy. Death puts life in perspective as we finally see it as the very fragile, easily lost, and infinitely valuable thing that it is. When you do not sense that death is after you, you relax and think yourself out of danger. But you have no guarantee that you are alive in five minutes. It is already later than you think. In a little while we are gone. In a moment we have all turned to dust.

To live with the awareness of death — death is around somewhere, waiting for our final slip in order to sweep us away — is a sinister but also wonderful situation. When we are aware of death and know that we have too little time left and that time is the only thing we do not have, then we really do our best. When we acknowledge the unique opportunity we have to become aware, straighten our lives to get a better life while there is still a chance, then we can live the way that makes each day better than the previous and the next year better than this. We can live in such a way that we are on our way up.

Ask yourself: “Am I on my way up or on my way down?” Do you have to admit that you are on your way down, even though of course it is a slow descent? The only thing that can make most of us change our course, so that we live in a manner that leads us upwards, is the distinct awareness of death. When we see death threatening us all the time and coming at us in many various forms like loneliness, illness, or hopelessness and when we realize that we constantly feed death with great chunks of our own flesh, because we do not make the right choices and thus unconsciously take one step further towards the grave, then we are motivated to correct these systematic faults.

Only death has the power to really make us want to change our course in life. All lesser problems and crises throughout life may be unpleasant, but not really unpleasant enough to make us want to succeed in changing ourselves. The reason for this extreme conservatism is that we already have dedicated most of our decisions to survive, i.e., to avoid dying. Therefore, the death that threatens us now is computed in our minds as more important than the death threatening us in the past. But awareness of death does not come to us easily when we are only slowly decaying. We can see people die in front of us without understanding that we, too, consist of fragile flesh and that we have to depart soon. No force in life can change this: In a little while we are gone. We have to live here and now. This moment is all we have got.

## WHEN YOU HAVE ONLY 700 DAYS LEFT

When “terminal” cancer patients (the quotation marks are because maybe the patients are not as terminal as we usually think) visit their physician some time after they have received the diagnosis and the verdict, that according to the statistics they only have about two years left to live, they often say strange things like: “I am grateful that I got cancer.” The physician thinks that this is strange and asks why. “I have never felt so well,” the patient says. Most physicians tend to think that is peculiar, because here we have Mrs. Larsen, who lost 35 kilogram, lost all her hair because of chemotherapy, and her cancer has metastasized throughout the body. Also she looks like something the cat dragged in. “But it is true, doctor,” she insists. “My life has never had so much meaning, my life is more intense than it has ever been now that I know that I have only 700 days left. Now I have let go on all my worries and idiosyncrasies. I have turned simple. I see the sun rise, I feel the wind on my skin, I talk honestly with my friends and I have stopped arguing with my husband. And best of all I have started to say no in order to only do the things I really like”.

However absurd it may seem, people with their back against the wall who know their days are numbered often live much more intensely than the rest of us, who imagine that we will live forever. Face to face with death we suddenly appear to remember that this is what life is all about, to feel good within ourselves and with each other and to do something we really like. “What a fool I have been” people often say and think. Time possesses the strange capacity to expand enormously, when we live intensely. A moment can feel like eternity or a year can appear to pass within a minute. (Have you ever experienced a New Year's Eve where you feel that the past year has been uneventful, so totally empty of anything essential?) If you know that you only have a short while left before death, then even such a moment may be enough to change the course that fate had in store for you.

We may call this very strange power that steps in when you are facing death "the will to live". We really do possess enormous potentials for growth and change, but only rarely do these potentials come into use. Our reason and our total naiveté towards the tough and wonderful conditions that apply to us prevent us from changing. You can reclaim the meaning of life. You can break through to the experience of being totally and fully alive, to your life having meaning and your existence making a real difference to the world you live in and yourself. Patients who become well again, drug addicts who become clean, prostitutes who succeed in love, all the miracles people talk about, but do not believe in. All this happens during this process. But, of course, up to this day it has been rare. If our culture held more insight into these things they would probably be much more common, the way they appear to have been in other cultures at other times. A holistic physician often has the great fortune to live in a world where these miracles are almost normal. In our "quality of life as medicine" projects they occur surprisingly often[12,13].

## TO PULL YOURSELF UP BY THE ROOTS OF YOUR HAIR

A force stronger than reason is needed when the course of your life is to be altered. Let us call it the will to a better life. If this will is present people will possess real humility, making them open and willing to learn and change. The strange thing about this will is that it is an irrational, nonverbal force that pulls up your existence.

When the will influences your view of the world it becomes altered in a strange way. This happens because the will to live supplies a fixed point, namely what you have to believe in when you really love life, beyond all reason, beyond everything you have learned and experienced in life. This fixed point can serve as a new foundation for your personal philosophy of life. From this moment on you will feel that deep down life is good and valuable, the world is full of opportunities, people are trustworthy, you are able to solve the problems in life on your own, and through this battle you can make everything cohere.

The experience of pulling yourself up by the roots of your hair literally means that you raise or lift your own existence. You correct your faults and close up all the holes that drain your vital energy. You remove all the good reasons for not having any self-respect and start a new life on a totally new foundation. You take responsibility for your own life.

The essential part of the will is that it is able to cut through all the confusion and doubt that normally characterize human life. In reality, there is no rational way of determining the truth value of statements or philosophies of life. You cannot guess the truth about life and the world. Reason cannot distinguish very well between personal philosophies of life, because they all basically rest on principles that are irrational, ethical, or even emotional. However, the will to live a good life cuts cleanly through doubt and mental fog and points out clearly and directly what is right and wrong in relation to our love of life.

Some decisions and choices are in harmony with life, others bring ruin and destruction. Some decisions lead towards the top, others towards the bottom. Some views of the world can sustain life, others weigh it down. Only the will to live a good life can make a person rise above the immaterial, the meaningless, doubt and nonsense. You rarely discover that the will is the real resource for improving life, until you are facing death. When that happens, the will to live is often the only reason why you survive.

The experience of pulling yourself up by the roots of your hair is quite amusing. But really, if we are to live fully and completely for just a moment this is what is needed, the ability to lift ourselves and take wings, despite the thousands of weights that are dragging us down. Taking responsibility for our own lives is really a process during which we elevate our own existence, in spite of all barriers and difficulties. The will to live a good life is the only thing that can create this effect.

## TO FIND THE QUALITY OF LIFE

We are free to choose our values, the things we think are important and good. Some people's lives are centered on collecting stamps, while others collect good friends. Some choose expensive clothes or fast cars as values, while others grow ecological vegetables and wear only clothes made of recycled material. Some people collect dirty videos, while others are into bible studies. In our minds we are free to choose our own personal values, just as we have an enormous freedom to describe the world whatever way we want.

One thing is values, another thing is how we feel or what state we are in. Something makes one person happy, something else makes another person happy. But what about the happiness we feel: Is it the same kind of happiness or are there different kinds of happiness? And what about satisfaction with life? Do we all possess the same sense of satisfaction or do we experience satisfaction in different ways? What about the meaning of life itself? When we feel deep within ourselves, in our very souls and hearts (if we are able to find it), do we then feel the same meaningfulness in life, when it is meaningful and the same senselessness, when it is not? Do two people experience the same kind of love, the same feeling of hate, or sexual desire?

It is obvious that each experience carries its own qualities and intensity. But is the actual quality of the experience connected to the individual, to our egos, and learned descriptions of the world? Or is the actual quality of happiness, satisfaction, or the meaning of life something that is given by human nature? As it appears from this paper, we believe very strongly in nature and that life within us never has let go of its habitat in nature, because we possess our common description of the world not just as a possibility, but as a necessity. This is a given, because we are constructed the way we are and have to live together.

The decisive factor for being able to change yourself is that you are able to regain your belief, that fundamentally life is good. When we here use the word "belief" it is because from a rational point of view, such an attitude will always be a question of belief. Subjectively, of course, it can also be experienced as certain knowledge. And it is this inner certain conviction that makes the difference.

You can believe nature as being the essential thing. Not, as is often suggested, in a primitive way, with coarse instincts and preprogrammed behavior parallel to animal behavior, but more refined. The idea is that deep down in our biological matter we possess a nature as humans. This nature is in the shape of an abstract recipe for being a human, and life is about expressing this recipe to the full, unfolding and manifesting its potential for a good life.

In this light, our nature holds the potential for all the dimensions of our lives. It is in our nature to feel good or bad, to be satisfied or dissatisfied, to have sexual feelings, to be happy and feel there is a meaning in life, or to work for our innermost visions and longings. Our nature is such that we have a heart that we need to discern and obey so that we may lead the good life.

## TO SEIZE THE MEANING OF LIFE

When we finally acknowledge that the world extends beyond our reason or that there are forces at large that matter more than our impulses, then we can proceed. When we realize that there are values on Earth that far surpass the value of our small life, then we will be humble enough to accept the gift (and task) that is life. Then we can put our faith in authority and our loyal, but out-of-date and limited, description of the world behind us.

To rediscover the meaning of life means finding yourself and the values that you can always, and without faltering, use as foundation for your own life. To regain the meaning of life means that you acknowledge that you are a human being, subject to the conditions and laws applicable to humans. We are not talking about the highway code, but more profound laws that apply to all living beings. To take responsibility, to see yourself as active and not as a victim, to work at correcting your personal faults, and repair the bumps in your inner map of the world.

To regain the meaning of life does not mean to be forever happy. It means that you find your fundamental challenge as a human being and take up the challenge. You become a person with a mission. There are things to be corrected both on the inside and the outside, things within yourself and things in the world around you. If you are really clever you will see that in reality there is often little difference between the two. The flaws in the world are evident to you, because you also sense and work on similar weaknesses and flaws within yourself. The great struggle for a better world, that all people become involved in, when they acknowledge that the meaning of life is about coherence. They cannot escape this world, however much they want to, because of all its superficiality, materialism, abuse of power, and false values, then they realize that this struggle is very much about improving that part of the world that is you. To clean the place you occupy, to cultivate your own spirit.

Everything starts with yourself. Because all the barriers you see are actually within you, in your own personal view of the world. Becoming free means first and foremost becoming free of the constraints imposed by your own rational description of reality. It does not actually mean that you must get rid of this description or the framework it puts around your self-expression, but you can loosen the constraints so much that they no longer limit life, but support life. You need to get rid of the negativity in the description and the old pains that hold your limiting decisions in their place.

## WHAT IS THE PURPOSE OF YOUR LIFE?

Imagine that you really wish to know the meaning of your life. You rent a small cabin in the mountains, where nobody can disturb you for the next three or four weeks. You buy provisions for the whole stay. You go alone and you spend your time on only one thing, namely answering the question: “What is the meaning of my life”? Of course you have to find a wording that is all your own, which exactly fits you and your life. But it must be deep enough to penetrate all the way into your soul.

What is the purpose of life for you? Why are you on the surface of the Earth for a short while? In what way do you make a difference in the world? What are your dreams in life, love, real friendship, a good job, or harmony with nature?

When you compare the life you lead with your dreams, how do you measure up? Is your personal relationship the love of your life or is it boring routine in bed and arguments at breakfast and before the evening news? Do you actually have one single friend with whom you can and do talk about everything and who does not begrudge you real progress? A friend who can meet you right where you are and just wish you all the best and therefore ask you all the questions you should already have asked yourself, but did not dare to out of fear of meeting yourself? Questions like what is it you want, what are your opportunities, and what is needed for you to obtain what you want with the opportunities you have?

What about your work? Do you really exert yourself and improve anything? Do you gain the expertise necessary to express yourself creatively and spontaneously? Do you solve your tasks to your own personal satisfaction? Do you have enough influence on your own work? Do you actually accept what your company produces or should you be doing something quite different in order to be of use in the world?

What about your time off? Do your holidays fulfill your dreams or do you just end up in some bar in Mallorca wasting your time on casual pursuits, before returning home to your boring routine? Do you burn for your life, your work, and your love? Does you or your life contain any nerve at all? In the final analysis, how do you feel if you are really and totally honest? Are you OK? Do you get out of life what it can give you? Do you exploit all your opportunities? Have you accepted the challenge that is yours and is your life in balance? Are you at peace with yourself, because you have acknowledged your own personal mission in life?

We suspect that after a couple of days you are having no more fun at the cabin. After all, to study the meaning of life is rather unpleasant. The really sad truth is that we have no wish to know the truth about ourselves or the deeper meaning of life, because it is painful to learn something decidedly new and we only do this if it is absolutely necessary.

Actually, what began as a straightaway philosophical experiment now appears to be a dramatic process, where you have to confront and process the pains of a lifetime! All the bad things you have done since early childhood will come to you and ask you for a clean-up! This is not our favorite perspective, but the only perspective that will make us change into better, more innocent and more loving persons.

## THE PAIN OF KNOWING THE MEANING OF YOUR LIFE

Our problem is that deep down we do not really want to know the meaning of our lives, because if we do we have to acknowledge that the life we actually live is a pale shadow of the opportunities we hold, no matter how good life is, when compared to that of other people.

We are not at all interested in realizing that we almost live in an existential gutter, when we compare our life with what we were actually created for. Our life is not first class and maybe it is not even second class, which we thought, but actually third class, because our life is more or less without love to life, to other people or even to ourselves.

We are also not the decent folks we thought we were, but rather harbor fairly violent and destructive tendencies. Not a fun perspective at all. Let us assure you that one of us was surprised when, one sunny day some years ago, he finally came to the realization that his basic intentions toward other people were mean, while he himself thought he was such a well-meaning fellow. A close examination showed otherwise. There is a reason why we do not want to know ourselves: It hurts. This realization that our life does not have the meaning it could have or that our life is far poorer than it needs to be does not give us a nice feeling. That we are actually at fault for wasting our life and perhaps about to lose something precious, our actual existence, this realization is actually very unpleasant.

The unpleasantness lies in the realization of the magnitude of the problem, because it obliges us to do something about it for our own sake. We must take responsibility and see ourselves as the cause of our own personal mess. We must learn to associate with others and change our attitudes towards all kinds of things. We need to let go of all out-dated points of view for which we have fought and battled forever, ever since we learned them from our parents.

It is important that you can face yourself in the mirror every morning. One reason this may be difficult is the painful feeling that you are not faithful to yourself. When you know deep down what life is about and what your real purpose and meaning of life are, it hurts inside if you just continue living as always and not true to your own intuitions.

When you are conscious of your big dream, but shy away from working to make it come true, you suppress yourself. This works fine only as long as you are not too aware of it, but with the growing awareness the suppression of your own life becomes still harder to bear. The more you understand the game of life, the more you are obliged to engage in it. Knowing what you like makes it much more difficult not to be good to yourself. When you face yourself in the mirror, you will know how much work you have to do to bring your life in better accord with the innermost wishes of your soul.

Everybody who engages totally in the challenge of improving his or her relationship with the self will find that this game can be won. It takes a real effort, though. For most of us, it is hard work every day for many years. Frankly because our state of being is so lousy when we start out. We are rather far from being happy, cheerful, and easygoing.

One of us with the experience of this process felt a strong and almost unbearable sensation of unworthiness. When you develop an excellent inner standard of existence, you are likely to feel less proud of yourself. When you realize the brilliant standard that all mankind inhabits deep down in his soul[4] — all that we are meant to be, our real potential — then our present existence often seems pretty pale, insignificant, sometimes close to a total failure. As long as you compare yourself with your next-door neighbor you can always claim success. But when your start comparing your present state of being with that of a person at his full peek — like Moses, Buddha, Jesus, Leonardo da Vinci, or maybe spiritual masters like the Baal Shem Tov, Dalai Lama, or Sai Baba — it is difficult not to feel gray.

Now, humility and humor will always be helpful. It is quite funny to be an action hero, when you compare yourself to your friends, and be an existential midget, when you compare yourself to your own potentials. You might find that what nature or God intended you to be is amazingly different from whatever you thought at first. The gift of knowing the meaning of life is energy. When we see our true potential it is tempting to reach for the power and glory, the creativity and the divinity that lie within. When we do this we will immediately get all kinds of problems with the outer world and we will get an immense amount of energy. An unsurpassed energy kick.

People who know their hidden potentials and dig into them without hesitation or second thoughts will always blossom. They will soon be transformed into original beings, colorful, intelligent, troublesome, creative, lovely, and often annoying like hell. These people will normally get everything they want. If they are sick they will get healed, if they are artists they will get fame, if they are scientists they will get a unique understanding of their field of research. Eventually, as their personal growth continues they might be recognized as the geniuses of this world.

The secret of these success stories is lots and lots of energy drawn from the source of existence combined with other amazing qualities like intuitive competence and emotional intelligence. These qualities pour from one single source: life. More precisely, the abundant source of energy and motivation is “the joy of life”. Joyfulness seems to be the most basic and most mysterious quality of all living beings. The nature of joy is by the way still completely unexplained by science.

## THE NO MAN’S LAND BETWEEN YOUR OLD AND YOUR NEW LIFE

Knowing what life is about does not necessarily mean that life becomes any easier. It is often quite the opposite: Life turns even more difficult when you wake up. But a conscious life has a peculiar quality. A person who experiences the deepest meaning in his or her life discovers that life now has a touch of bliss and fragrance[14]. No matter how chaotic, no matter how painful, deep down the new life is sweet.

This fine sweetness makes it possible for a human being to endure almost incredible pain and sorrow. When you strive to realize yourself and your utopian dreams, many people will react as if you have the plague. You will often turn into an incomprehensible and disturbing element of other people’s worlds. To be sure, after some years of hard work you will come back as a beautiful, peaceful, and happy person, but often the first thing that happens is that you turn annoying, selfish, difficult, or even angry.

The fine, inner sweetness gives these people an unstoppable quality. They turn into fighters. They have seen the light and they follow it. New jobs, divorces, new friends, new habits and values, new sexual and professional interests... we are talking about major transformations here. People are not the same and will never be the same again. They are forever lost for you, if you do not follow them by developing yourself.

If you get a metastasized cancer and you heal yourself by letting go of the negative beliefs and self-suppressing decisions of a lifetime, you will be changed. You now have dramatically improved your quality of life and inner coherence. But you might also be in the situation where you find yourself as reborn to the degree that not even your old clothing fits you any more. The price to be paid for personal growth is, unfortunately, chaos. As most people are very conservative, they will try to oppose your growth the best they can. So, people who supported you when you were down suddenly do their best to suppress you. It is sometimes difficult to believe that your relatives can jump on your back trying to hold you back. It is sometimes grotesque that you will have to escape from your whole family.

Between your new blossoming life and the old normal, boring life of habits and routines is a no man’s land of very difficult nature. You discover that nothing is as you thought is was — it might be that your beloved does not really love you or that what you thought was the essence of your life is simply a substitution for a sound and healthy interest. Often people going through this transformation will at some point in time feel that they are going crazy. Relax, you are not going crazy. You have been crazy for half a lifetime living with values that did not make you happy. And now you are waking up. You are in the middle of a speedy, but unpleasant recovery. Loneliness of the most painful kind is normal at this stage. You are alone with your thoughts, and you are confused, unhappy, not seen, not loved, and not understood.

You cannot continue to live your old life, but you have not yet found your new ways. The fine order of your life has been broken and now chaos prevails both on the surface and in the debts of your soul.

We are healing, but first we must acknowledge that we really are sick. The pain of a whole lifetime is often overwhelming and survival becomes dependent on our ability to be good to ourselves. Nobody but yourself is there now to show you love and concern. The miracle is that it is enough: When we love ourselves we do not really depend on other people's concern for us. But before we can enjoy the luxury of relying fully on ourselves, living in perfect inner balance, we must heal a lot of old painful wounds. This is why loneliness bites us at this stage.

Most people live lives that are not truly a life. They sense this intuitively, but they do not want to look at it at all. There are plenty of symptoms telling you that everything is not as it is supposed to be. The terrible headache or low back pain that returns still more often, problems sleeping at night, the growing sexual problems that are taking the fun out of this part of life, problems with your skin, the slips of memory, maybe the arthritis making every step you take even more painful. Enough is enough. Some day you realize that this is not how you want to live. Enough of lies and politeness and pretension. Air! You need fresh air, renewal, new inspiration. The way you live brings you slow death and this is not how life was meant to be. It takes a lot of courage to break the well-known order of daily life.

Sometimes we are lucky enough to be forced to make the move and wake up; the physician gives you the malignant diagnosis, the boss says he is sorry he has to let you go, because of your still poorer performance. This is the end. You have reached the end of the road. You only have one chance now: renewal from within. Your whole life needs repair. It is time to clean up the mess. Now a hard time usually follows. It is difficult not to feel that a lot of time is wasted living your old life. Realizing the distance to the existence you have been living, you are often overwhelmed with sorrow and bitter regrets. But eventually you will find mercy and realize that life is never wasted, you have learned your lesson, you suffered for as long as you had to. As time goes by, you will appreciate a still deeper pattern of order and inherent logic in the universe.

## DISCUSSION

Several questions can be raised about the important, practical side of this philosophy[5,6,12,13,14]. Some of the problems are like paradoxes and the solutions are somewhat surprising. Let us investigate some of the hard problems, often dealt with in existential holistic therapy[14].

- How can we cultivate joy if a person has no energy to live? This seems impossible, but here is an example showing how it can be done.

One of the authors (SV) was called to see a female patient, 48 years old, with metastasized cancer of the cervix. Before she became ill she had been a strong, attractive, beautiful, healthy, and successful woman. After 41 sessions of radiation, her pelvis was scared from the radiation burns to such a degree that she was now completely exhausted and almost dying. She was severely immune-suppressed and had, in spite of antibiotic treatment, infections in the mouth and infections in her bladder. She had lost weight — from 70 to below 50 kg — had a low hemoglobin with severe pains from her pelvis, back, and neck whenever she was moving. She had absolutely no appetite and wanted to die after the physicians had told her that the cancer was still in her cervix and maybe also in her liver. She could not turn her head to talk to me. She could hardly get out of her bed by herself. So, what to do?

I asked myself the question: Do you love her? And I did. So I told her. Then I thought she looked terrible and I also told her that. She smiled and then I realized that she wanted to die. I asked her if that was true. It was. So I saw her and I understood her. Then I touched her on her burned stomach and explained to her that if she wanted to live, she had to come back with her awareness into her pelvis and confront the pain. She did that, while I kept my hand on her. She cried and I cared for her. She was sad, because she had lost her hair. So I accepted her exactly as she was. She felt ashamed. I acknowledged that and allowed her to have her feeling. After an hour she was glad. She was almost happy. A few days later she came home from the hospital and was able to take her first walk. Maybe she will die. But I made her glad.

So do not think about it. Quality of life philosophy is not really for your head. It is for living, loving, and doing good. There are always hidden resources. You go find them!

- What values should be endorsed according to the quality of life philosophy? This looks like a wise question as values are guiding so much of our behavior. But the quality of life philosophy is not as much a product of the brain — *thinking of values* — as a product of heart and intuition — *feeling the value.* Let us look a little closer on this difference.

Life is not really about *values*. Life is more abstract, like it *is the only valuable* thing. So life is *the one value* that should be celebrated and taken into focus. If you split life up in a number of subvalues, you might miss the whole point. Life is in flow always, so go with the flow. Be intelligent, be smart, be wise, be strong, but before anything… be loving. Life is something special and different to everybody. For some life is joy, for others life is understanding, for others again life is care and tenderness. The important thing is to let life find its own expression in every person you meet. If you can see the unique color of life in the person in front of you, you guide this person towards his or her true nature. Being oneself is also knowing your purpose of life, and this purpose carries all the meaning and motivation that makes us able to take responsibility for our own suffering.

So practicing the quality of life philosophy is not about thinking, it is not about values, it is about being human, and allowing yourself to meet your patient as one whole human being and connecting to another whole being.

- The authors appear to contrast a “materialistic” culture with a “spiritual” culture. Can these two cultures be combined? This also looks like a paradox, but is not.

Philosophically our world view is neither materialistic nor spiritualistic, but in between. In our opinion life exists in a continuum, which looks like matter from one end and like spirit from the other end, but is neither. Life is characterized by structure, joy, and consciousness. These three qualities seem to be aspects of the biological information[14], which seems to be associated with anything living, in such a complex way that completely new laws of nature must be discovered before we can give a reasonably adequate account of the living state. Most likely some form of complicated quantum chemistry and a whole new concept of physical energy is needed. This information can even guide the organization of whole societies of organisms, just look at the ants. The central point here is that the biological information can also guide human beings, although it probably happens in a far more dynamic way, that is, through human consciousness. This looks a lot like a spiritual approach, but it is so important not to forget the body and the life in the body.

Researchers and physicians on the other hand tend to reduce all living organisms to chemistry and only focus on certain chemical aspects of these organisms. However practical it is to know these chemical aspects, it seems rather trivial if compared with the living organism’s highly advanced processing of the biological information.

Life is built up through a large number of levels, with the still higher levels possessing ever more complex and abstract traits, including the qualities known so well in the human world: love, hate, consciousness, the subconscious, reason, and intuition. But consciousness is not more important than the material world. We believe that matter and consciousness are of exactly the same importance for life.

## CONCLUSION

We have a nature as human beings. It is this nature that makes it possible for us to be happy, cheerful, wise, and lovely. When we turn natural and innocent, the extraordinary freedom that characterizes life at its fullest will return to us. All the life we hold as living organisms will now blossom and grow. Our love and passion will come back, a burning interest for our work will unexpectedly catch us, deep friendships will form, and this divine creativity and humor will mark our new personality. All of us have the possibility to make a difference. The quality of our own life can be drastically improved and so can our use to people around us[15]. We can be of real value to ourselves and to the world around us. Instead of being one more of these human beings tearing down the global ecosystem, you will understand the web of life in all its forms and shadows and do what is needed to make mankind and our beautiful culture survive.

As we see it, mankind is a highly endangered species, and only by transforming our old materialistic culture into a new spiritual culture with honesty, truthfulness, and contributing people can humanity survive. The right place for all of us to begin is by saving ourselves. All it takes is that we decide to seize the meaning of life. But this must be one whole-hearted move: You must give it everything you have got if you want to succeed. You can change a poor life to an excellent life[1,2,3,4,7,8,16], but you must risk your life to win. One day you will find the courage. Maybe the day is today. The wise Jewish Rabbis have a few good sayings: “Live today as if tomorrow is your last day” or “in order to perfect yourself, one must renew oneself day by day”.

Let us conclude by telling the story of Sol Gordon, professor emeritus of Child and Family Studies at Syracuse University: “Growing up as an idealistic youth, I was determined to save the world and even the more I tried, the world became worse and worse. Then I decided I had taken on too much. I thought I would just try to save the United States. The more I tried — conditions in the US got worse and worse. So again I thought I had taken on too much. So I decided I would just try to save my neighborhood. My neighbors told me to mind my own business. But just as I was about to give up in despair, I read in the Talmud (Jewish teachings) that if you can save one life, it is as though you have saved the world. That is now my mission — one person at a time”[17].

## ACKNOWLEDGMENTS

This study was supported by grants from The 1991 Pharmacy Foundation, as well as by supplementary grants from Goodwill-fonden, the JL-Foundation, E. Danielsen and Wife's Foundation, Emmerick Meyer's Trust, the Frimodt-Heineken Foundation, the Hede Nielsen Family Foundation, Petrus Andersens Fond, Wholesaler C.P. Frederiksens Study Trust, Else & Mogens Wedell-Wedellsborg's Foundation and IMK Almene Fond. We gratefully acknowledge the critical scrutiny and expert linguistic assistance of Ib Ravn, Ph.D. and also the assistance of the philosopher Maximilian Kroman. The research was approved by the Copenhagen Scientific Ethical Committee under number (KF)V.100.2123/91.

## SELECTED READINGS FOR THE PATIENTS

Our patients have benefited from reading the following small books and others, which in our opinion express a clear and positive quality of life philosophy, not very different from the quality of life philosophy presented here.

- Bach, R. (1970) *Jonathan Livingston Seagull.* Macmillan, New York.
- Chopra, D. (2000) *How to Know God*. Harmony Books, New York.
- Exupéry, A. (2000) *The Little Prince*. Harvest Book, New York.
- Gibran, K. (1971) *The Prophet.* Alfred A. Knopf, New York.
- Hesse, H. (1982) *Siddhartha.* Bantam Books, New York.

## BIOSKETCHES

**Søren Ventegodt, MD,** is the Director of the Quality of Life Research Center in Copenhagen, Denmark. He is also responsible for a Research Clinic for Holistic Medicine in Copenhagen and is a popular speaker throughout Scandinavia. He has published numerous scientific or popular articles and a number of books on holistic medicine, quality of life, and quality of working life. His most important scientific contributions are the comprehensive SEQOL questionnaire, the very short QoL5 questionnaire, the integrated QOL theory, the holistic process theory, the life mission theory, and the Danish Quality of Life Research Survey, 1991–94 in cooperation with the University Hospital of Copenhagen and the late pediatric professor Bengt Zachau-Christiansen. E-mail: ventegodt@livskvalitet.org. Website: www.livskvalitet.org/

**Niels Jørgen Andersen, MSc,** Professor, Department of Innovation and Economic Organization, Norwegian School of Management. This department conducts research and provides teaching in central topics related to innovation, business development, management of global companies, business history, and economic organization. Research activities within the Department are related to four core subjects within the discipline: business history, cooperative organizations, business development and entrepreneurship, and finally studies of industries with a special focus on the electricity industry. He is also the dynamic chairman of the nonprofit organization Stiftelsen Holistisk Medisin Scandinavia, which aims to support the scientific development, research, and documentation of complementary and holistic medicine in Scandinavia. E-mail: niels.j.andersen@bi.no. Website: www.bi.no/users/fgl93013/

**Joav Merrick, MD, DMSc,** is Professor of Child Health and Human Development affiliated with the Zusman Child Development Center and Division of Community Health at the Ben Gurion University, Beer-Sheva, Israel and presently the Medical Director of the Division for Mental Retardation, Ministry of Social Affairs, Jerusalem and the Director of the National Institute of Child Health and Human Development. He has numerous publications in the field of child and human development, rehabilitation, intellectual disability, disability, health, welfare, abuse, advocacy and prevention. Dr. Merrick received the Peter Sabroe Child Award for outstanding work on behalf of Danish Children in 1985 and the International LEGO-Prize ("The Children's Nobel Prize") for an extraordinary contribution towards improvement in child welfare and well being in 1987. E-mail: jmerrick@internet-zahav.net. Website: www.nichd-israel.com
